# Enhancing carbon emission reduction strategies using OCO and ICOS data

**DOI:** 10.1038/s41598-025-22022-1

**Published:** 2025-10-17

**Authors:** Oskar Åström, Carina Geldhauser, Markus Grillitsch, Ola Hall, Alexandros Sopasakis

**Affiliations:** 1 Centre for Mathematical Sciences, Lund University, 22362 Lund, Sweden; 2 Munich Center for Machine Learning, Garching, 85748 Germany; 3 Department of Mathematics, ETH Zurich, 8092 Zurich, Switzerland; 4 Human Geography, Lund University, 22362 Lund, Sweden

**Keywords:** Attribution, Climate and Earth system modelling, Climate-change policy, Applied mathematics, Computer science

## Abstract

We propose a methodology to enhance local CO_2_ monitoring by integrating satellite data from the Orbiting Carbon Observatories (OCO-2 and OCO-3) with ground-level observations from the Integrated Carbon Observation System (ICOS) and weather data from the ECMWF Reanalysis v5 (ERA5). Unlike traditional methods that downsample national data, our approach uses multimodal data fusion for high-resolution CO_2_ estimations. We employ weighted K-nearest neighbor (KNN) interpolation with machine learning models to predict ground-level CO_2_ from satellite measurements, achieving a Root Mean Squared Error of 3.58 ppm. Our results show the effectiveness of integrating diverse data sources in capturing local emission patterns, highlighting the value of high-resolution atmospheric transport models. The developed model improves the granularity of CO_2_ monitoring, providing precise insights for targeted carbon mitigation strategies, and represents a novel application of neural networks and KNN in environmental monitoring, adaptable to various regions and temporal scales.

## Introduction

In this era of rapid climate change, the precise monitoring of carbon dioxide emissions has become a critical task for governing bodies and environmental organizations worldwide. The ability to accurately assess and manage CO_2_ levels is not just a scientific challenge, but a necessity for effective climate action.

The current landscape of carbon dioxide ($\hbox {CO}_2$) measurement is fraught with challenges, predominantly due to the lack of comprehensive and localized data. Local authorities, which are on the front line of implementing climate action strategies, often find themselves relying on national-level data or extrapolations from limited ground measurements. This lack of granularity in the data can lead to inaccuracies in identifying local emission sources while also impeding the development of more effective, targeted climate strategies.

Our research aims to bridge this gap by using satellite-derived data to provide a more precise and localized view of CO_2_ emissions. We adopt a multimodal data approach, utilizing data from the Orbiting Carbon Observatories (OCO-2 and OCO-3) as well as the Integrated Carbon Observation System (ICOS) and the ECMWF Reanalysis v5 (ERA5) model, enabling us to establish vital connections between satellite transects, ground-level CO_2_ data, and weather. This integration is pivotal in enhancing the precision and reliability of CO_2_ datasets derived from satellite observations, offering an unprecedented level of detail in emission monitoring.

In technical terms, our aim is to build a machine learning algorithm which, using satellite data for carbon dioxide, weather, and other relevant input factors, can model accurate measurements, similar to ICOS ground data measurements of the biospheric surface CO_2_ flux anomalies.

However, monitoring of CO_2_ using satellite data is not without its challenges. Unlike pollutants with relatively short atmospheric lifetimes such as $\hbox {NO}_2$ and $\hbox {SO}_2$, CO_2_ has a longer lifespan, resulting in a high background concentration that complicates the identification of new anthropogenic sources. Furthermore, the spatiotemporal distribution of CO_2_ is heavily influenced by atmospheric dynamics and terrestrial biospheric fluxes, introducing significant variability and seasonality in measurements.

Although significant strides have been made in atmospheric CO_2_ monitoring, the complexity of the task remains, with challenges in regions of complex topography and varying meteorological conditions. Continuous advancements in satellite technology, data processing, and atmospheric modeling are crucial to enhance the accuracy and scope of global CO_2_ monitoring.

Our methodology addresses these challenges by creating machine learning models that employ advanced statistical analyses to estimate ground observations of CO_2_ from satellite data. We also explore the utility of high-resolution satellite data in capturing small-scale variability of $\hbox {CO}_2$, which is often lost in coarser-resolution models. This approach is critical in understanding the nuances of CO_2_ weather - the interaction between weather patterns and CO_2_ surface fluxes – and in providing accurate data for regional modeling studies and field experiments.

We begin in Section “State of the art” with an overview of satellite-based CO_2_ monitoring approaches and their utilization in CO_2_ estimation. Then, in Section “OCO-2 and OCO-3: advances and challenges in CO_2_ Monitoring” we describe current methodological advances as well as challenges in the data collected from the OCO-2 and OCO-3 satellites. We then describe in more detail the methods and data used in this work in Section “Materials and methods”. We present our findings and end with a feature importance analysis of the models in Section “Results”.

## State of the art

In this section, we provide an overview of key satellite instruments that have significantly advanced the field of CO_2_ monitoring. We discuss the specific contributions of satellites such as SCIAMACHY, GOSAT, and OCO-2, highlighting their roles in improving the precision of CO_2_ measurements and their applications in environmental research. The discussion includes an analysis of how these satellites have been utilized to detect CO_2_ emissions from various sources, understand the effects of atmospheric transport on CO_2_ distribution, and enhance our overall understanding of the global carbon cycle.

In recent decades, significant advances have been made in satellite technology for environmental monitoring. Notable among these are instruments like SCIAMACHY (Scanning Imaging Absorption Spectrometer for Atmospheric Chartography) onboard the Environmental Satellite, ENVISAT, operational from 2002 to 2012, and the Greenhouse Gases Observing Satellite (GOSAT), launched in 2009. These satellites, along with others such as the American OCO-2, the Chinese TanSat, and the upcoming Geostationary Carbon Observatory (GeoCarb), have revolutionized our ability to monitor atmospheric $\hbox {CO}_2$. Each of these satellites offers unique contributions to the field, from high-precision CO_2_ observing capabilities to the monitoring of CO_2_ variations on seasonal time scales.

The analysis of data collected by SCIAMACHY has been instrumental in deriving empirical regional conversion factors, which are used to estimate CO_2_ emissions based on observed NO_2_ columns. This approach, as demonstrated in previous studies^1,2^, helps establish a crucial link between NO_2_—a short-lived pollutant indicative of fossil fuel combustion—and the longer-lived CO_2_, thereby enhancing our understanding of anthropogenic emissions and their impact on atmospheric composition. SCIAMACHY data was also used to estimate CO_2_ abundance from fossil fuel emissions, particularly from power plants^3^. This approach has provided insights into the spatial distribution of CO_2_ emissions, contributing to a more nuanced understanding of anthropogenic impacts on atmospheric CO_2_ levels.

Greenhouse gases observing satellite (GOSAT) observations have been pivotal in detecting CO_2_ emission signatures from urban centers like Los Angeles and Mumbai^4,5^. This detection capability is crucial for understanding the role of megacities in global CO_2_ emissions. These satellite-based CO_2_ abundances can be compared with emission inventories, revealing potential gaps in emission estimates, as was shown in a study from East Asia^6^.

The capabilities of OCO-2 surpass those of earlier instruments such as SCIAMACHY and GOSAT, particularly due to a significant reduction in xCO_2_ retrieval uncertainty. This reduction is achieved through OCO-2’s higher spectral resolution, advanced data processing algorithms, and improved spatial and temporal coverage. As a result, OCO-2 provides more precise and reliable measurements of CO_2_, enhancing our ability to monitor global emissions, validate emission inventories, and improve climate models, thereby deepening our understanding of CO_2_’s role in climate change.

Feldman et al.^7^ showed that OCO-2 can also detect anomalies arising from terrestrial biosphere extremes, for example droughts and heat waves with a rate of 80% in some parts of Northern Australia and ’greater than by chance’ detection rates for the most extreme CO_2_ surface flux anomalies in western US (^7^, page 1555). This advancement is crucial for detecting subtle CO_2_ anomalies that may be indicative of significant environmental events or trends.

### Challenges in CO_2_ emissions estimation

Carbon dioxide presents unique challenges due to its longer atmospheric lifespan and high background concentration, which complicates the identification of anthropogenic sources^3,8,9^. The bottom-up compilation of CO_2_ inventories, based on reported data or human activity such as energy consumption and fuel purity, faces notable discrepancies in emission estimates. These uncertainties are especially pronounced in megacities in developing countries^10,11^.

Due to its longer lifespan, atmospheric transport of CO_2_ becomes a relevant factor, which can lead to overestimation in deserts and underestimation in areas with significant vegetation, as indicated by He et al.^12^. Their study highlights how extreme changes in atmospheric CO_2_ concentrations, detected using satellite data, are influenced by local environmental conditions and transport dynamics, underscoring the complexity of accurately estimating CO_2_ levels in diverse regions.

The variability of atmospheric CO_2_ is heavily influenced by its interaction with weather patterns, which requires high-resolution atmospheric transport models. These models are critical for capturing small-scale variability and understanding the intricacies of CO_2_ weather^7^. However, monitoring global carbon sources and sinks is highly complex and needs to quantify longer latency and errors due to assumptions about uncertain surface CO_2_ flux drivers and meteorological conditions^7^.

The integration of weather data, as explored by Hakkarainen et al.^8^, Beirle et al.^13^, and Fioletov et al.^14^, offers a more comprehensive understanding of emissions, particularly from isolated urban areas. Incorporating wind speed information is crucial for interpreting emission patterns and understanding the dynamics of pollutant dispersion. Research indicates the need for undisturbed atmospheric transport for accurate xCO_2_ retrievals, highlighting the need for low variability in wind direction and speed, a consistent wind source, and the avoidance of complex topography near the surface^7,8,13,14^.

### OCO-2 and OCO-3: advances and challenges in CO_2_ monitoring

The Orbiting Carbon Observatory satellites, OCO-2 and OCO-3, represent a significant advancement in the global monitoring of carbon dioxide (CO_2_). Unlike direct measurement techniques, these satellites detect CO_2_ by measuring the absorption of sunlight reflected from the Earth’s surface within an air column^15,16^. This approach, combined with their high sensitivity, enables these satellites to provide detailed insights into atmospheric CO_2_ concentrations. The measurement area for OCO-2 and OCO-3 is approximately $1.29 \times 2.25$ km, achieved through a sun-synchronous orbit that ensures consistent data acquisition across different regions^7^.

A key strength of OCO-2 and OCO-3 lies in their ability to generate column-averaged dry-air mole fractions of CO_2_. This is accomplished through advanced retrieval algorithms, which integrate point measurements to produce highly accurate data for further analysis^17^. The precision of these satellites has been instrumental in urban emission monitoring, particularly in evaluating fossil fuel CO_2_ emissions from densely populated areas. High-resolution transport modeling, combined with Bayesian inversion systems, has been used to optimize city-wide emission estimates^18^. Furthermore, the Snapshot Area Mapping (SAM) mode of OCO-3 has proven effective in characterizing major anthropogenic sources, especially when supplemented with $\hbox {NO}_2$ measurements from other satellite instruments^8^.

However, despite these advancements, OCO-2 and OCO-3 face several challenges that limit their effectiveness in certain scenarios. Firstly, their sun-synchronous orbits and narrow measurement swaths restrict the frequency and coverage of observations over specific regions, especially concerning point sources such as power plants, where emission origins are concentrated to a single location. Additionally, both satellites are subject to retrieval errors and observation gaps, which can hinder the consistent detection of flux anomalies, particularly in complex terrains or under specific atmospheric conditions^16^. The accuracy of data can also be affected by biases introduced through small pointing errors, especially in regions with rough topography. Therefore, rigorous data filtering and bias correction, calibrated against standards like TCCON and the WMO CO_2_ reference scale, are crucial to maintaining data integrity^17^.

The modeling of point sources, such as coal power plants^7^, further highlights the challenges associated with satellite-based CO_2_ monitoring. For instance, several studies^19–21^ have shown that a significant proportion of satellite data can be rendered unusable due to unfavorable atmospheric conditions, such as cloud cover, aerosols, or haze, which obscure the satellite’s view and lead to retrieval errors. Even when data retrieval is successful, such measurements may fail to capture information near or even downwind of the emission source due to, for instance, sudden changes in wind speed and misalignment between the emission source and the detected plume^7^. These factors underscore the need for continuous refinement of satellite-based CO_2_ monitoring techniques in order to improve data reliability and accuracy.

While OCO-2 and OCO-3 have significantly advanced our capability to monitor global CO_2_ emissions, ongoing challenges in data retrieval, particularly over complex terrains and smaller emission sources, underscore the need for continued refinement in both satellite technology and data processing methodologies.

## Materials and methods

This study uses data from multiple openly available high-resolution datasets, providing comprehensive measurements of the CO_2_ concentration and the atmospheric variable, which we describe in the following section.

### Datasets

We began by employing OCO-2 and OCO-3 satellite data consisting of bias-corrected, retrospective xCO_2_ measurements from the Level 2 mission, available at https://disc.gsfc.nasa.gov/datasets/OCO2_L2_Lite_FP_10r/summary. To ensure accuracy and reliability, this data is pre-processed at the source by the OCO-2 and OCO-3 Science Teams at NASA and associated institutions^22^. This pre-processing also includes physics-based algorithms to adjust for systematic errors through bias correction and produce improved measurements of the mole fraction of CO_2_ in dry air, as determined from the sun absorption spectra in the near-infrared at 1.61 and 2.06 $\mu$m. The satellite data, characterized by a spatial resolution within a one-hour time frame, covers a parallelogram-shaped area of 2.29 km$^2$ per column with an orbit track width of 10.3 km. Due to the slow nature of API requests, the retrieval of weather data was performed on a one-degree latitude and longitude grid and restricted to a six-hour window from 9:00 to 15:00.

Second, we utilized ICOS station data, incorporating measurements from 32 ICOS atmospheric stations across Europe since 2014. These stations provide hourly averages of dry-air mole fractions of $\hbox {CO}_2$. They employ nondispersive infrared (NDIR) analyzers for the spectral analysis of air samples. Unlike the OCO dataset, ICOS data are geographically fixed and offer continuous time series data. However, it is important to note that the recordings are not always complete due to various factors such as instrument malfunctions, power failures, extreme weather conditions, and regular maintenance, which result in interrupted data at most stations.

Third, we used ERA5 Reanalysis Weather Data, sourced from the Copernicus programme and the ECMWF’s Climate Data Store. This dataset provided supplemental weather data, including wind components at 10*m* height, surface-level pressure, and temperature at surface-level and 2m height. The additional variables of humidity, cloud coverage, soil water content, convective energy, and total precipitation were also included. In total, this resulted in 10 weather features. These weather features, along with the OCO measurements, time, latitude, and longitude, resulted in an input vector of size 14.

The study period spanned from the beginning of the OCO mission in September 2014 to March 2023. The spatial coverage for the weather data for OCO, ICOS, and ERA5 was defined within a geodesic rectangle bounded by coordinates $28^\circ S$, $-17^\circ W$, $70^\circ N$, and $64^\circ E$. The sun-synchronous orbit of the OCO satellite resulted in more frequent data collection over European ICOS stations during summer and reduced coverage in winter.

To construct the dataset, satellite-based $\hbox {CO}_2$ measurements from the Orbiting Carbon Observatories were temporally and spatially aligned with ground-based observations from the nearest ICOS station. Specifically, an OCO measurement was included only if a corresponding ICOS observation was available within a ±30-minute window. Given that ICOS stations record data at hourly intervals, this matching criterion typically allowed for reliable pairing, except in cases of missing or invalid ICOS data due to station downtime or technical issues.

Although satellite overpasses are limited to specific orbital windows, this matching strategy yielded a substantial dataset comprising 38,623 matched OCO–ICOS pairs spanning the years 2014–2023. Of these, 31,039 were used for training and 7584 for spatially independent validation. This represents a sizeable and representative sample of conditions under which both surface and satellite observations are available. Thus, while the matched data represent a subset of all possible OCO and ICOS observations, the resulting dataset is sufficient in scale and diversity to support robust model training and validation.

**Fig. 1:**
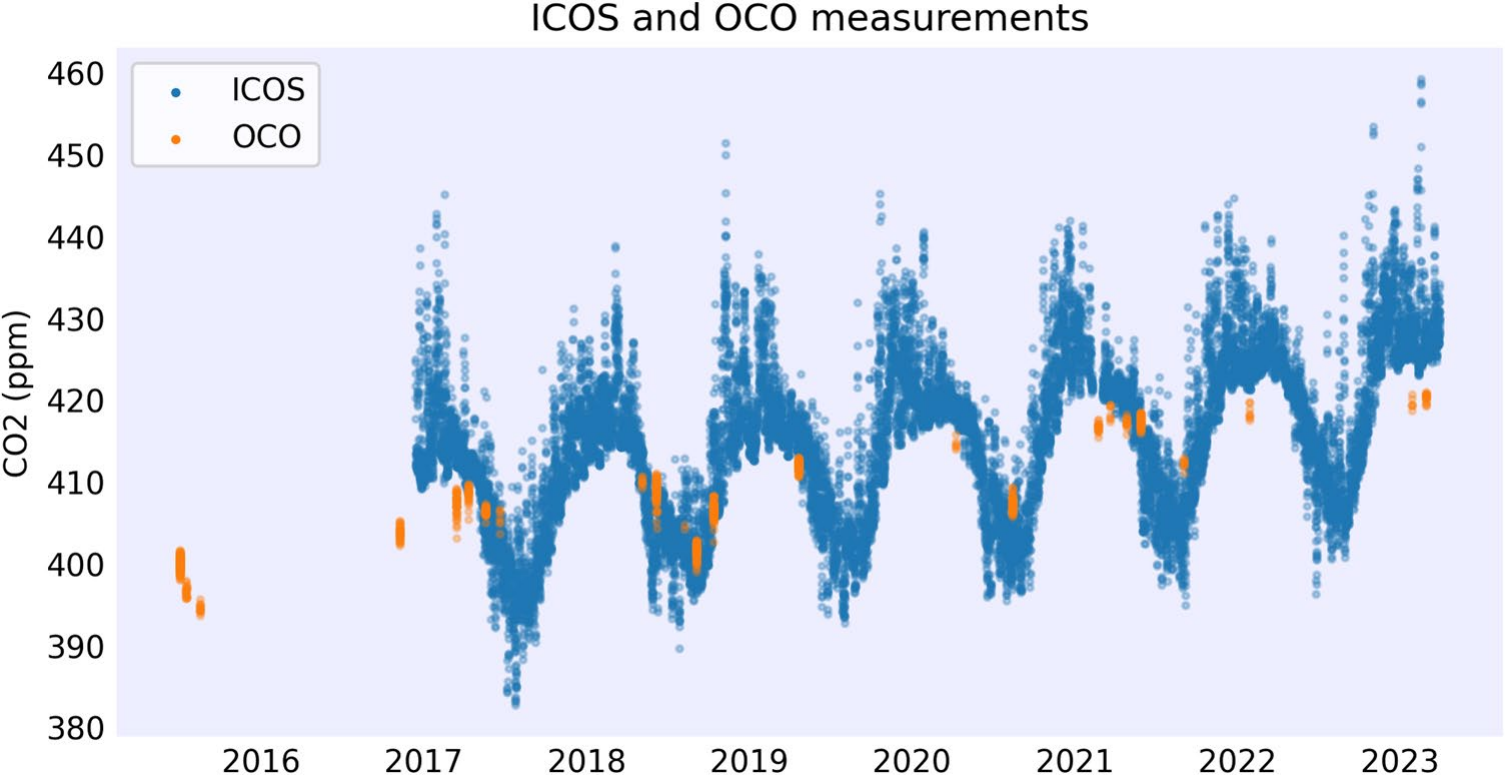
Measurements from the Hyltemossa ICOS station (blue) and nearby OCO observations (orange) within a 25 km radius for the time frame of data availability. ICOS measures near-surface $\hbox {CO}_2$ while OCO provides column-averaged $\hbox {CO}_2$ ($\hbox {XCO}_2$).

Figure 1 illustrates this sparsity in the vicinity of the Hyltemossa ICOS station. Despite fundamental differences in the measurement modalities—OCO reporting column-averaged dry-air mole fractions of $\hbox {CO}_2$ ($\hbox {XCO}_2$) and ICOS capturing near-surface concentrations—the seasonal trends observed in the two datasets are broadly consistent, suggesting a coherent temporal pattern across atmospheric layers.

To increase the variance in the input data, the OCO measurements were augmented with ERA5 weather data and the location and time information of the measurement. ICOS stations record weather in addition to CO_2_. However, since these stations are geographically fixed and primarily record wind speed and direction, using ICOS data as input prohibits the use of this model outside of the station bounds. Consequently, ERA5 data was used for a more comprehensive weather analysis as it has global reach and records more weather features.

### Machine learning models

To estimate CO_2_ levels from satellite data, we used multiple classes of machine learning models.

As a baseline, we perform simple linear regression on ICOS measurements based only on OCO observations. This baseline, therefore, does not contain any weather data and is a measurement of how well the OCO satellite data itself correlates with the ground-level CO_2_.

Secondly, we employ Boosting models, chosen due to their proven efficacy in handling complex non-linear data relationships, which is characteristic of environmental datasets^23,24^. Boosting models in general are a type of ensemble machine learning method designed to improve the accuracy of predictions by combining the outputs of multiple weak learners into a single strong model. The key idea behind boosting is to sequentially train weak models, where each model focuses on correcting the errors made by the previous models in the sequence. The ensemble of weak models is hence improved by iteratively adding new models that are trained to predict the pseudo-residuals of the previous iteration of the ensemble. These models have been shown to outperform classical methods like Stochastic Gradient Descent and Support Vector Machines in environmental monitoring^24^.

We employed two different types of boosting models: Category Boosting (CB) and Extreme Gradient Boosting (XGB). XGB is a boosting model that uses continuous input features to describe the data, whereas CB employs categorical features and outputs. This discretizes the data into less descriptive but more complex classes, rather than a point in N-dimensional space. The parameters and packages used for CB and XGB are specified in the Supplementary Material.

In addition to employing Boosting models, we utilized a multilayer perceptron (MLP) neural network tailored for regression tasks. This MLP comprises five densely connected hidden layers, encompassing a total of 23,809 trainable parameters—a relatively modest architecture by contemporary standards. This compact model was deliberately chosen due to the nature of the dataset, which includes 14 features that exhibit strong correlations with the ground truth. Furthermore, since the measurements were exclusively obtained from locations surrounding the ICOS stations, a more complex model would likely lead to significant overfitting to the training data. Specific details on the model’s architecture and implementation can be found in the Supplementary Material.

Each model was trained on the same dataset of 31,039 data points and evaluated on a test set of 7584 data points. The test data came from four ICOS stations that were deliberately excluded from training. These stations were randomly selected and geographically distant from one another to ensure a diverse and spatially independent evaluation set. This design helps prevent data leakage—a common issue in spatial modeling that can compromise model validity. Hyperparameters for each model were optimized via a traditional grid search. This step is particularly important for boosting models, which are known to be sensitive to hyperparameter settings^25^. Fine-tuning mitigates the risk of regression collapse and improves overall performance. Final model configurations are provided in the Supplementary Material.

## Results

In this section, we present the outcomes of our machine learning models applied to satellite and ground-based data for estimating ground-level CO_2_ concentrations. We first compare the performance of different models, including the CB, XGB, and MLP, in terms of their accuracy and robustness. This is followed by an analysis of multi-scale CO_2_ predictions, extending beyond the ICOS station data to larger geographic areas. Finally, we evaluate the significance of various input features using SHAP analysis, providing insights into the key drivers of the model’s predictions.

### Ground-Level CO_2_ Estimation

To perform the ground-level CO_2_ estimation, we used the baseline model, CB, XGB, and MLP as described in Section “Machine learning models”.

The baseline model achieved a Root Mean Squared Error (RMSE) of 6.21. This is a measure of the amount of error in the predictions, and a low RMSE is desirable. The CB model exhibited an RMSE of 4.35, while the XGB model achieved an RMSE of 4.04. Finally, the MLP achieved an RMSE of only 3.58. Table 1 presents a comprehensive comparison of these models, including the additional statistical metrics of Mean Squared Error (MSE) and Adjusted R2, providing a holistic view of model performance. In addition, the table shows $95\%$ confidence intervals for these metrics. Confidence intervals were estimated using bootstrapping with 10,000 resamples. The results suggest that the MLP is able to capture more of the variability in the data. These metrics are calculated using

1 $$\begin{aligned} \begin{array}{c} \text {MSE} = \frac{1}{n}\sum\limits_{i=1}^{n}(y_i - {\hat{y}}_i)^2, \hspace{0.3cm} \text {RMSE} = \sqrt{\text{ MSE }}, \\ R^2_{\text {adj}}=1-\frac{n-1}{n-m-1}\cdot \frac{\displaystyle \sum\nolimits_{i=1}^{n}(y_i - {\hat{y}}_i)^2}{\displaystyle \sum\nolimits_{i=1}^{n}(y_i - {\bar{y}})^2}, \end{array} \end{aligned}$$

where *n* denotes the number of data points in the test set, *m* is the number of input features, $y_i$ represents the true ICOS measurement, ${\hat{y}}_i$ is the model prediction, and ${\bar{y}}$ is the mean of the ground truth values. The MSE quantifies the average of the squared differences between predicted and actual values, while the RMSE provides an interpretable metric in the same units as the target variable (ppm $\hbox {CO}_2$). The adjusted $R^2$ measures the proportion of variance explained by the model while penalizing for the number of features used, thus accounting for model complexity.

The input feature vector used for training and evaluation consists of 14 variables: (i) the satellite-derived $\hbox {xCO}_2$ value from OCO-2 or OCO-3, (ii) 10 weather-related variables from the ERA5 reanalysis dataset (including wind speed and direction at 10 m, surface pressure, 2 m and surface-level temperatures, relative humidity, cloud cover, soil water content, convective energy, and total precipitation), and (iii) spatial and temporal metadata—latitude, longitude, and timestamp. These features are described in more detail in Section Datasets.

**Table 1:** Model performance on the held-out test set using the evaluation metrics defined in Equation (1). Values are reported along with 95% confidence intervals, computed via bootstrapping (10,000 resamples).

Model	RMSE	MSE	Adjusted $R^2$
Baseline	6.21 ± 0.10	38.55 ± 1.24	0.517 ± 0.016
CB	4.35 ± 0.11	18.94 ± 0.93	0.787 ± 0.011
XGB	4.05 ± 0.10	16.43 ± 0.79	0.794 ± 0.009
MLP	3.58 ± 0.08	12.84 ± 0.59	0.839 ± 0.007

**Fig. 2:**
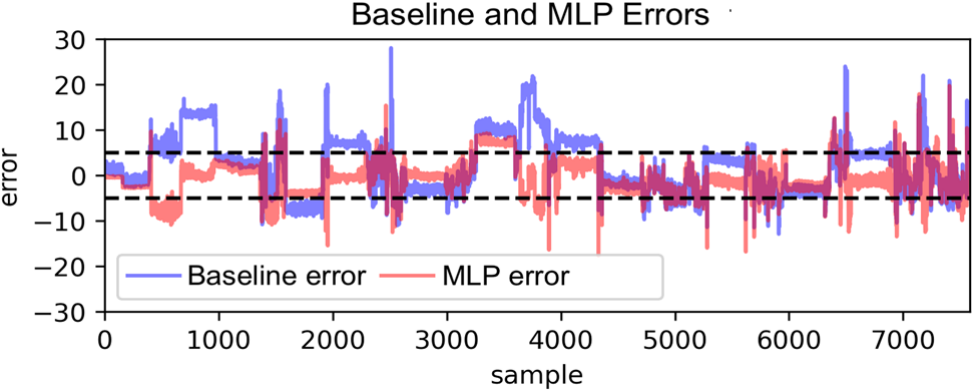
In blue, the difference between the actual ICOS and actual OCO measurements (Baseline error). In red, the difference between actual ICOS and predicted ICOS using the MLP model (MLP error). The dashed lines highlight that most of the MLP predicted values lie within an error of ±5ppm.

To tangibly compare the performance of the MLP to the baseline, Fig. 2 displays the prediction errors from the two respective models. From the results, it is clear that some patterns seen in the baseline are also visible in the MLP predictions. Mainly, Fig. 2 shows clear flat sections where prediction errors remain semi-constant in both models. These are satellite measurements that are close in space and time, and it is therefore natural that they result in similar predictions. However, it is also clear that the model is effectively able to shift the error of these sections closer to 0 using the additional contextual weather and location data provided.

**Fig. 3:**
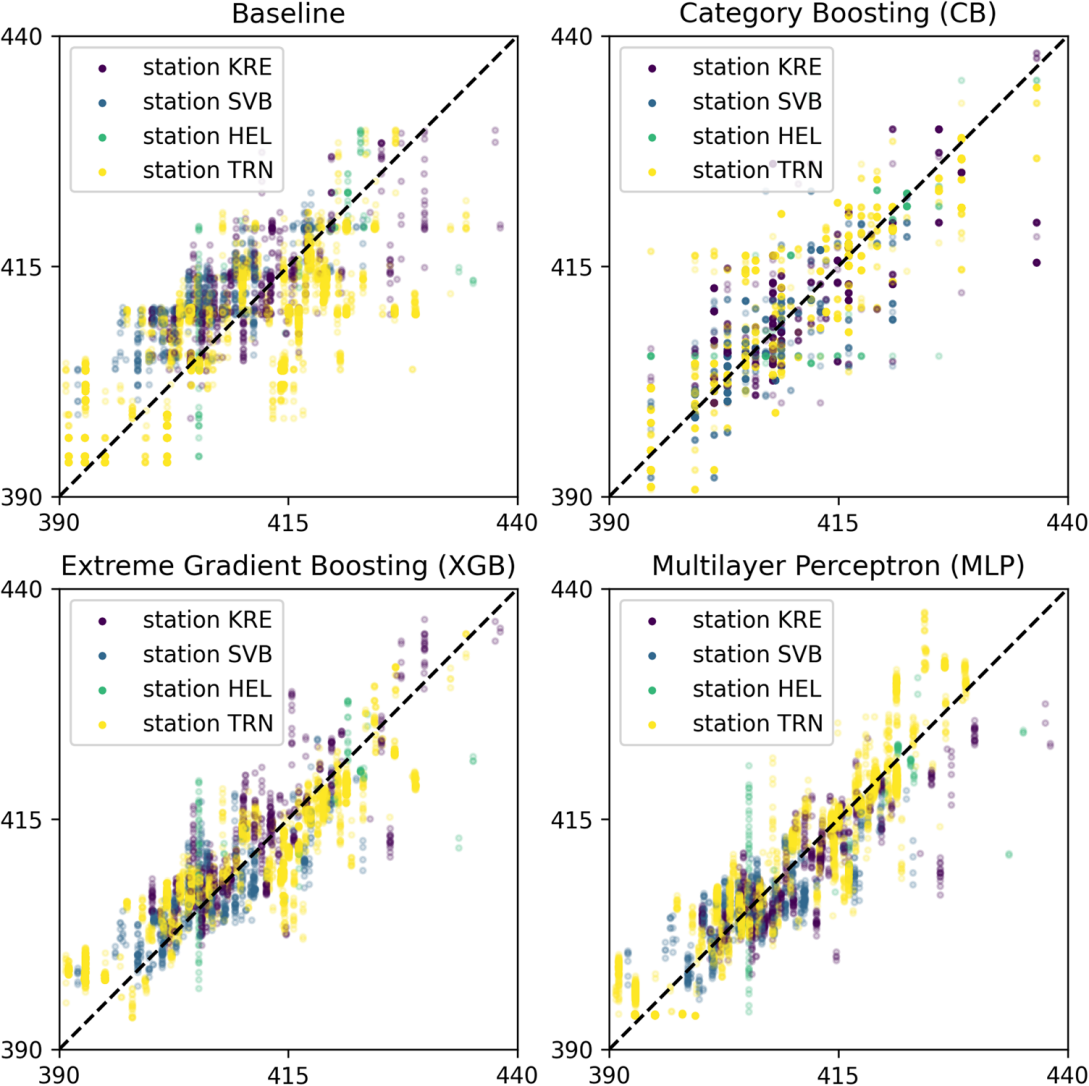
Correspondence plot between the predicted and true CO_2_ levels for the test dataset. The y-axis shows the predicted CO_2_ level from each respective model, while the x-axis shows the true ICOS measurement. The locations for each station’s acronym listed in the legend can be found at https://www.icos-cp.eu/observations/station-network.

In addition, Fig. 3 illustrates the predictions on the test set. We can infer from this figure that the MLP predictions are somewhat better aligned with the true ICOS measurements, as indicated by their proximity to the diagonal line as well as the lower RMSE score of Table 1. Furthermore, these results show that the residuals are of similar magnitude for both high and low CO_2_ levels. This suggests that there is no bias in prediction quality for extreme values. Moreover, the effects of the discretization in the CB model are apparent in the figure, where the predictions are constrained to a set of fixed values. This is a consequence of the architecture of the CB methods, which, instead of predicting a value for the ground-level CO_2_, classifies the measurement as belonging to one of a fixed set of possible values. This lowers the resolution of the predictions, but can sometimes be more effective for predictions. This was evidently not true in this case.

### Multi-scale predictions

The results above are based on ground truth measurements from ICOS stations, which have a limited geographical reach. However, since the predictive features rely solely on satellite information from ERA5 and OCO satellites, predictions do not need to be confined to just ICOS locations. To do this for a given region and timescale, OCO measurements were collected and combined with the corresponding weather information. These data points were used to make estimates of ground-level CO_2_ at each point. Since OCO doesn’t have full coverage every year, the available measurements had to be interpolated to neighboring locations. Note that since no ICOS measurements exist for these locations, these predictions cannot be verified against a ground truth. Instead, this section serves as an example of potential use cases of the methodology, should such ground truth measurements be available.

The interpolation of estimated CO_2_ levels was done via a weighted *K-nearest neighbor* (KNN) interpolation method, where the weight function was chosen to be $w_i=1/d^q$, where *d* is a spatiotemporal distance to the measurement and the parameter *q* corresponds to the decay in the weight function. This methodology estimates the value of CO_2_ at a given location and time, denoted here as $p^* =(x^*,y^*,t^*)$, by considering the predicted values at the *K* nearest spatial and temporal OCO measurements and assigning weights to them based on their distance from the point of interest.

Specifically, given a set of OCO observations at location and time $\{p_i\}_{i=1}^N=\{(x_i, y_i, t_i)\}_{i=1}^N$, and corresponding predictions $\{(v_i)\}_{i=1}^N$, the goal is to estimate the value $v^*$ at a new point $p^*=(x^*,y^*,t^*)$. This is done by first assigning each observation with a distance to the point of interest $p^*$, defined through,

2 $$\begin{aligned} d_i=d(p^*, p_i) = \sqrt{(x_i-x^*)^2 + (y_i-y^*)^2 + \sigma _t(t_i-t^*)^2}, \end{aligned}$$

where $\sigma _t$ is a scaling factor that translates a distance in time (days) to a corresponding distance in space (kilometers). The pseudocode of the proposed methodology is provided in Algorithm 1.

**Algorithm 1:**
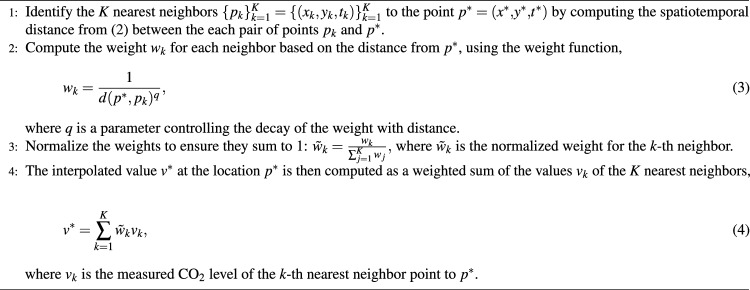
Interpolation procedure

**Fig. 4:**
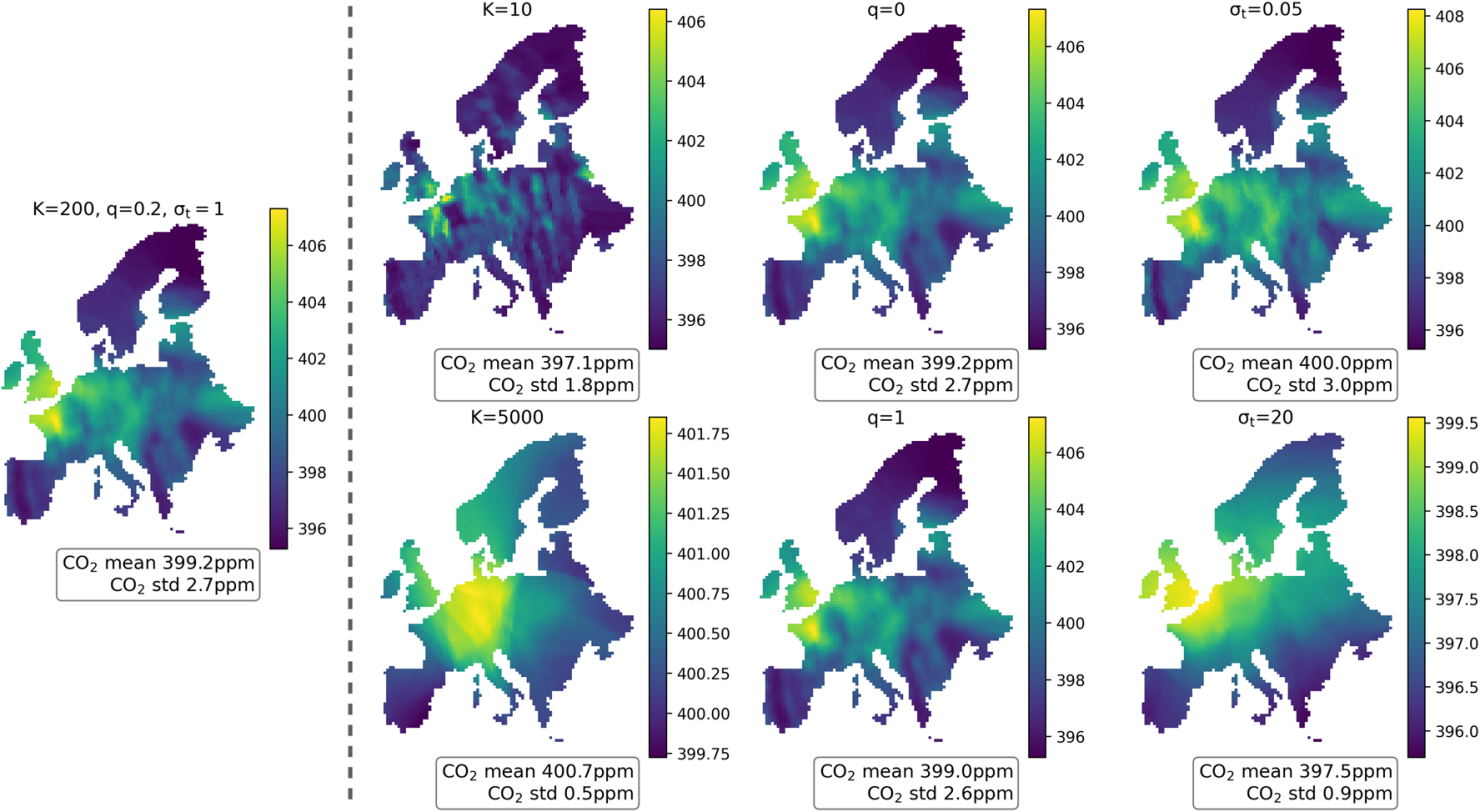
Interpolated predictions for Europe’s 2015 ground-level CO_2_ concentrations using different parameterizations of the weighted K-nearest neighbor interpolation. The ablation study shows results for the parameters $K=200$, $q=0.2$, and $\sigma _t=1$ to the left. For each parameter, the results for two surrounding values are presented. For each parameter configuration, the mean and standard deviation of the interpolation results are presented. See Algorithm 1 for details. *(Generated using the Python package Pyplot)*.

This methodology allows for a more flexible interpolation that adapts to the density and distribution of the measured data, with the parameters providing control over the smoothness and locality of the interpolation. The effect of these parameters can best be understood in the ablation study presented in Fig. 4. It was observed there that the average CO_2_ levels remained relatively constant and close to the mean of the measurements, regardless of the interpolation parameters. However, the standard deviation varied significantly between parameterizations, decreasing for large $\sigma _t$ and for extreme values of *K*.

The parameter *K* serves as a smoothing factor, where selecting a value that is too low introduces noise and artifacts associated with the satellite’s orbital patterns, while a value that is too high excessively smooths the data, leading to a loss of important local details in the predictions. The influence of the parameter *q* is relatively minor. We speculate this is because neighboring measurements are closely spaced and thus have similar distances. The time scaling $\sigma _t$ also behaves similarly to *K* in that it tends to smooth the results. The unit of $\sigma _t$ is *km*/*day* and corresponds to how many kilometers of distance one day delay is equivalent to. Too low of a $\sigma _t$ does leave some temporal artifacts and noise in the image, but a high one greatly reduces the detail of the predictions. Through empirical testing, we found that setting $K=200$, $q=0.2$, and $\sigma _t=1$ produced visually coherent results at the European scale, whereas $K=15$, $q=0.1$, and $\sigma _t=1$ yielded more detailed and salient estimates suitable for smaller, municipal-level analyses. It should be emphasized that, in the absence of extensive ground-truth validation data, these parameter choices remain heuristic. They are intended solely to support the illustrative regional visualizations presented in this section.

**Fig. 5:**
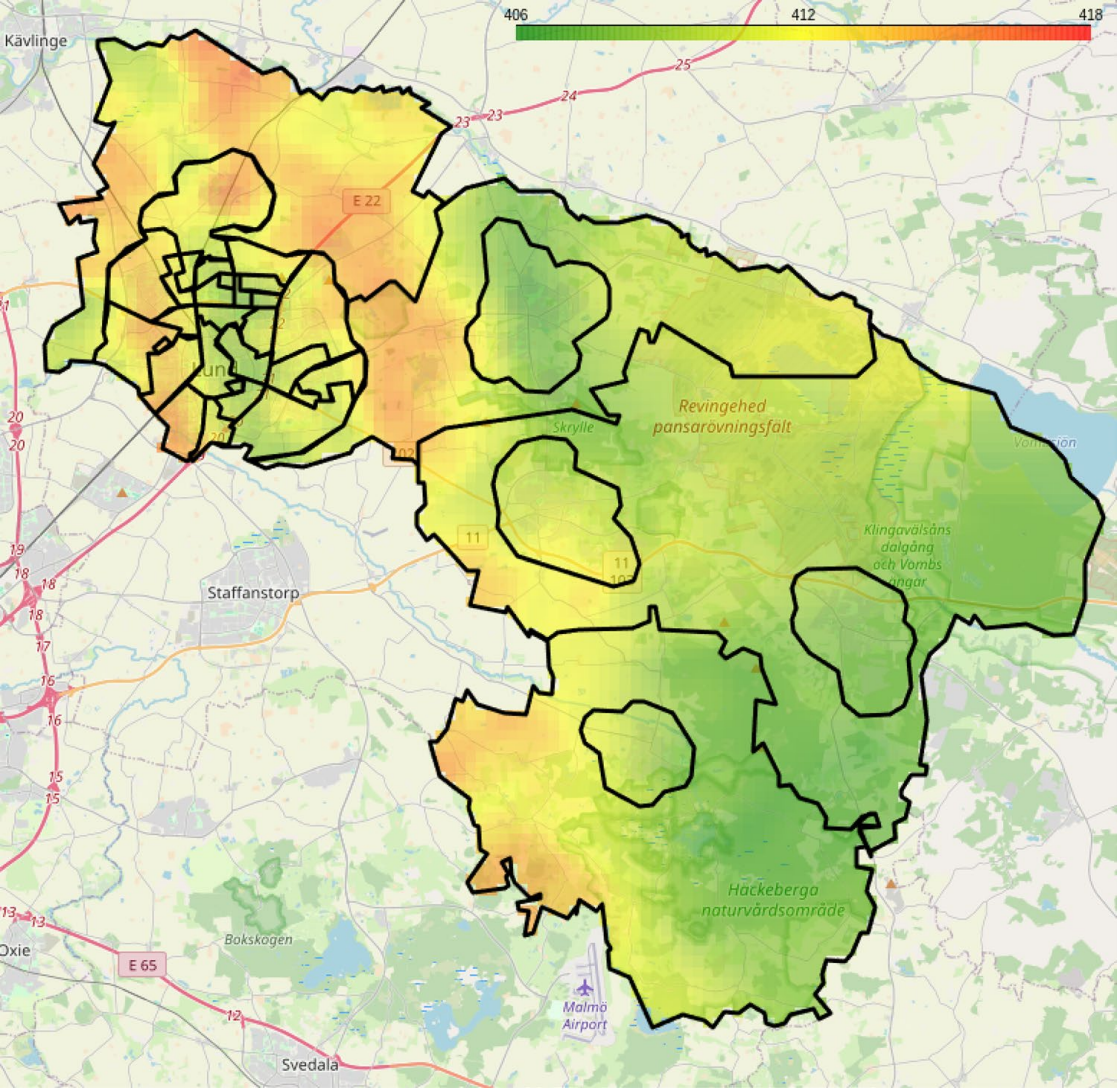
Predictions on the municipality of Lund, in southern Sweden, using the MLP model presented in Section “Machine learning models”. The KNN interpolation is made using $K=15$, $q=0.1$, and $\sigma _t=1$. The estimated CO_2_ levels are highest close to the city of Lund (top left) and further south near the Malmö Airport (center bottom), while they are lowest around the Häckeberga natural park reserve (middle right). *(Generated using the Python package Folium)*.

As a first example, Fig. 5 displays the average predicted ground-level CO_2_ levels over Lund Municipality. The highest level of ground-level CO_2_ is predicted to be around northern Lund, and the lowest around Häckeberga nature preserve. We do not have ground truth data here to compare to, but at a glance, this is a reasonable result as the preserve is reasonably a carbon sink. The higher levels to the north of Lund could possibly be due to the highway or perhaps the Örtofta sugar mill. Although it is important to stress that these results cannot be verified.

**Fig. 6:**
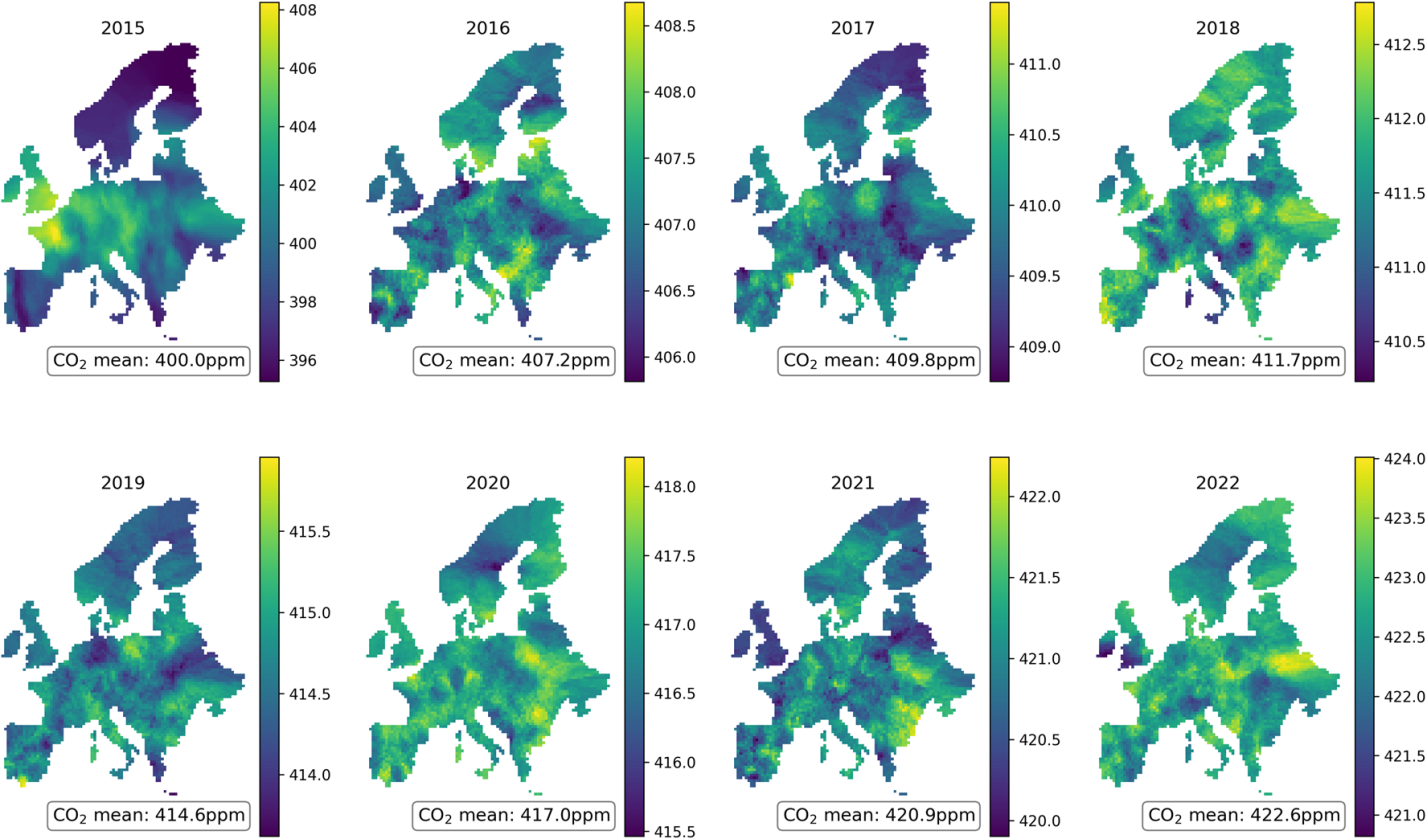
Annual ground-level CO_2_ estimations across Europe from 2015 to 2022, derived using the MLP model described in Section “Machine learning models”. The figure highlights spatial variations and temporal trends in CO_2_ concentrations over the entire region, showcasing the model’s capability to capture and predict CO_2_ levels at a high spatial resolution. The KNN interpolation was made using $K=200$, $q=0.2$, and $\sigma _t=1$. *(Generated using the Python package Pyplot)*.

The proposed approach has significant potential for scaling to larger regions, enabling the generation of high-resolution predictions across both time and space. Figure 6 illustrates yearly ground-level CO_2_ estimations across Europe, capturing the expected upward trend in mean CO_2_ concentrations over time. Notably, these predictions are produced at a high spatial resolution, even for locations where no ground stations currently measure CO_2_. Although the limited availability of ground-level CO_2_ data at such high spatial resolutions restricts our ability to independently validate these results, the figures highlight the model’s versatility. The model is capable of providing detailed predictions across varying spatial and temporal scales, surpassing the resolution of existing ground-level measurements.

### Feature importance

To analyze the importance of the OCO and weather features used in predictions, a SHAP analysis^26^ was employed. This method measures the change in output from the model when an input feature is changed. The feature importance is the amplitude of the prediction change when tweaking the corresponding feature.

**Fig. 7:**
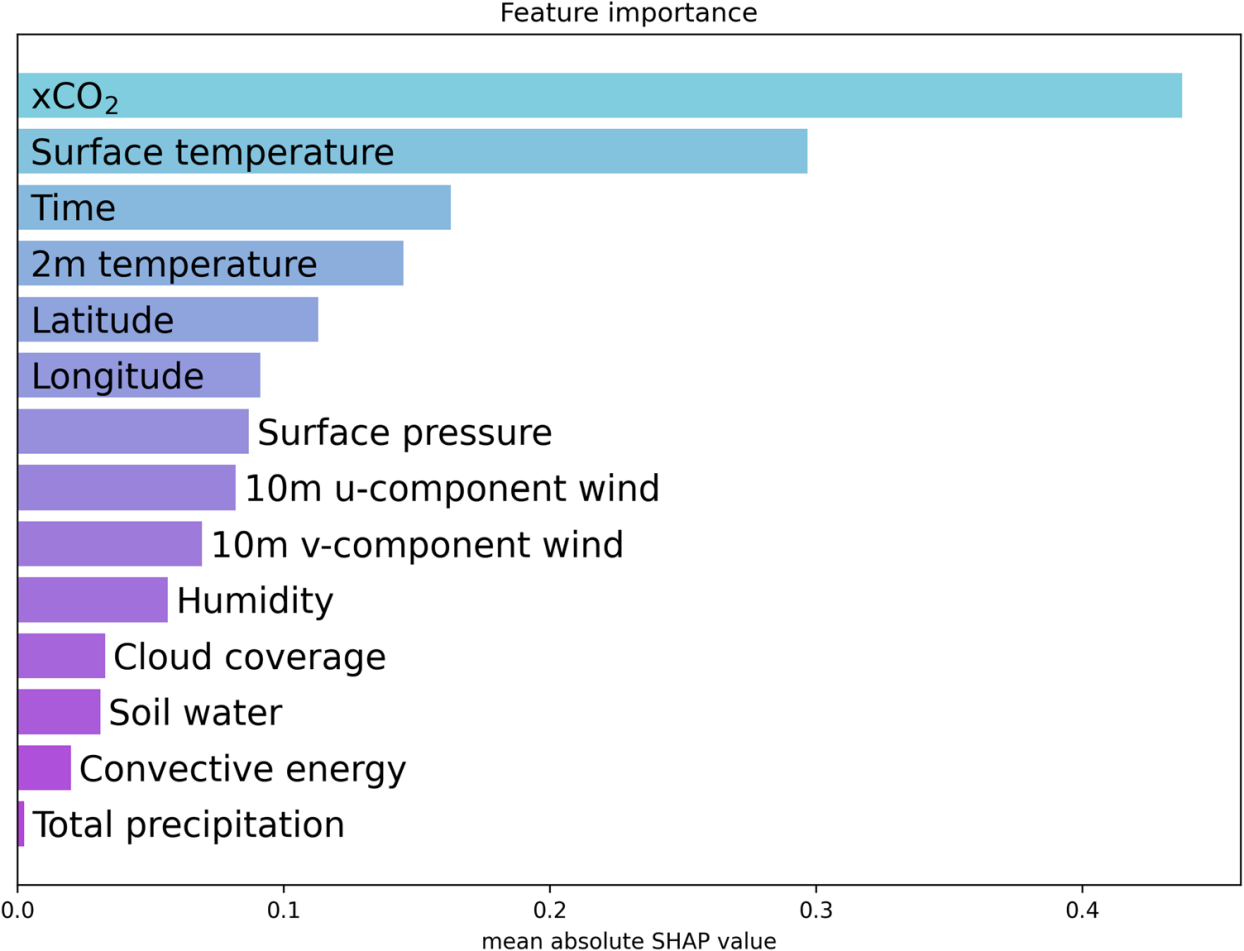
The importance of features in the input data using SHAP analysis based on the model results presented in Fig. 2. The three most important features were the xCO_2_ OCO measurements, surface temperature and time. The colors in the figure are used solely for differentiation and do not convey any additional information.

The results, shown in Fig. 7, highlight the relative importance of various features in our predictive model. The most significant feature identified was the $\hbox {xCO}_2$ from OCO2. This finding is expected, as $\hbox {xCO}_2$ provides a direct measurement of carbon concentration, making it a crucial indicator for our analysis.

In addition to $\hbox {xCO}_2$, temperature variables, specifically surface temperature and temperature at 2 meters, were also identified as important features. This result could be attributed to the fact that temperature serves as a proxy for the vertical movement of $\hbox {CO}_2$, influencing its distribution and concentration in the atmosphere, see^27,28^ for an overview. Moreover, the analysis revealed that geographic location (latitude and longitude) and temporal variables (time) are significant predictors. These features likely capture the global variations in atmospheric patterns that are not fully explained by other weather-related variables. Including location and time in the model helps account for the spatial and temporal heterogeneity in atmospheric $\hbox {CO}_2$ levels.

Overall, the SHAP analysis underscores the necessity of incorporating a diverse set of features, including direct measurements, proxy indicators, and spatial-temporal variables, to enhance the predictive accuracy of atmospheric $\hbox {CO}_2$ models.

## Discussion

The results indicate that we can achieve accurate predictions of ground-level CO_2_ with an RMSE of under 4ppm and an adjusted R2 value of over 80%. This can roughly be interpreted as the model typically guessing within 4ppm of the true value, and that the model can explain over 80% of the variation in ground-level $\hbox {CO}_2$. While the model achieves high accuracy on the task of predicting ICOS measurements, there are several paths for further improvement.

Firstly, while cloud coverage is included in the model, more detailed information about aerosol content is not included in the input features. This should not cause a bias in the model, as the ICOS-OCO data collection occurs regardless of aerosol content and should therefore result in a model suitable for all-sky conditions. However, including more detailed aerosol information could boost performance further.

Secondly, while point measurements of ERA5 wind are included, this only represents the current wind speed and direction. However, the xCO_2_ depends not only on the current winds, but how the wind has traveled and from where. Allowing the model to take into account the cumulative wind history in the area could allow for more accurate predictions. This could be achieved, for example, using a graph neural network^29^ over the ICOS locations or using a long short-term memory model^30^.

Since the predictive features only consist of satellite data, the model can be applied to locations outside of ICOS stations and is therefore able to render high-resolution predictions for any region. However, it is important to note that since ground truth data does not exist outside of ICOS stations, the extrapolated results cannot be verified at this moment.

The primary limitation of the current iteration of the model is the lack of good ground truth data. Since ground truth data is sparse and only localized around ICOS stations, the model will likely contain some biases towards these locations. Due to their size, stations are predominantly placed in rural areas or uninhabited regions, which means that there is very little ground truth data in cities or highly industrialized regions. In addition to this rural bias, we also have a continental bias as the ICOS stations are only located in Europe. The vast variety of landscapes, weather, and emission patterns that exist in the world cannot be said to be captured in the localized ground truth data available. Therefore, while the global reach of satellites allows for worldwide use of the model, it is highly discouraged at this stage. The primary application, for now, remains inside Europe.

That being said, the model can easily be adapted with additional ground truth data from any location in the world, thus opening up the possibility for global use in future iterations.

The second limitation is that while OCO has global reach, it doesn’t produce evenly distributed measurements either in space or time. Some regions, such as the south of Europe, receive many more observations compared to northern Europe due to their geographical location. Closer to the poles, the observations are sparser and only receive a few orbits per year. This means that weekly or monthly averages will be highly inaccurate for many locations. Therefore, analyses should ideally keep to yearly averages or larger time spans.

With this in mind, the current stage of the model is primarily suited for estimations in Europe of yearly averages in large-scale regions, or multi-year averages in smaller areas. The high-level resolution of the prediction could give additional insights into where emissions stem from and where the most important carbon sinks are located. To increase usability, future research should focus mainly on expanding the availability of good ground truth data. While the ICOS stations provide invaluable information with great resolution in time, they provide only part of the picture due to their limited reach. A dataset consisting of ground-level CO_2_ measurements with low time resolution but high spatial spread would be a great contribution to this field as a complement to ICOS. Incorporation of global ground-level CO_2_ measurements would also contribute greatly by ensuring global reach and availability of predictions.

This methodology of using remote sensing with verified ground truth data will be crucial for equitable and informed climate action due to the fact that manual emission testing or CO_2_ measuring stations are not an option for everyone. If this is done with steps ensuring that the predictions are unbiased and representative of most locations and conditions, we believe that this can be a vital tool for local efforts to mitigate climate change.

### Towards better climate action

The foundation of our research lies in addressing the significant gap in localized CO_2_ measurements, which hampers the effective design, implementation, and monitoring of precise local climate initiatives. The remote sensing approach we present has demonstrated its capacity to predict local CO_2_ levels, offering a promising alternative to this critical shortcoming. At the same time, we outline a clear pathway for further refinement of this approach, emphasizing the need to ensure that verified ground truth data is unbiased and representative of diverse global locations and conditions.

Given the currently available data, the model introduced in this article holds significant potential to enhance climate action efforts by enabling:

- Identification of High CO_2_ Concentration Areas: This allows for the strategic targeting of climate actions where they are most needed, optimizing resource allocation.
- Monitoring of Temporal Changes in CO_2_ Levels: This facilitates the assessment of climate action effectiveness over time, providing feedback that can guide future interventions.
- Effective Public Communication: By using visual tools such as maps and graphs, we can communicate CO_2_ level changes clearly to the public, fostering greater engagement and commitment to climate initiatives.
- Validation of Reported Emission Data: This contributes to the enforcement of environmental policies by verifying the accuracy of reported emissions, enhancing transparency and accountability.
- Support for Multi-Level Governance: Local administrations and European policymakers can rely on the same data sources, promoting coordinated and cohesive climate strategies across different governance levels.
- Integration with Socio-Economic Data: Combining local CO_2_ data with socio-economic indicators enables the analysis of synergies and trade-offs within socio-ecological systems, supporting more holistic decision-making.

Due to the limitations of verified ground truth available, the current model is particularly well-suited for use across Europe, providing valuable insights for large-scale regions (such as countries) based on yearly averages, as well as for smaller-scale regions (such as municipalities) when considering multi-year averages.

## Conclusions

Atmospheric CO_2_ monitoring has seen substantial improvements in recent years, driven by advancements in technology. However, significant challenges persist. The commonly used bottom-up approaches, which rely heavily on reported data, often suffer from biases and gaps in data coverage. Moreover, the long atmospheric lifespan of CO_2_ compared to other pollutants like methane and aerosols leads to extensive spatial distribution due to atmospheric transport. To address these challenges, the integration of detailed weather data with sophisticated atmospheric models is crucial. As satellite technology continues to evolve, it offers increasing potential for more precise and localized monitoring of CO_2_ emissions, which is critical for effective climate action and informed policy-making.

Our study aims to provide a more granular view of CO_2_ emissions to support policymakers in developing precise local climate action strategies. The proposed methodology goes beyond traditional approaches that rely on national data downsampling by leveraging multimodal data collection for more accurate results.

While the proposed methodology shows significant promise, it does have some limitations. A potential limitation of the proposed methodology is the sparse availability of ground truth $\hbox {CO}_2$ measurements, particularly in urban and industrial regions. The reliance on data from ICOS stations, which are primarily located in rural and European settings, may introduce biases, potentially underrepresenting CO_2_ variability in areas with higher emissions. Additionally, the OCO-2 and OCO-3 satellites do not provide uniform temporal and spatial coverage across all regions, particularly in areas with complex terrain or those distant from the equator. This uneven coverage could result in data gaps and impact the accuracy of CO_2_ estimations over time and space, particularly in regions with limited direct observations.

The integration of satellite data with ground measurements is shown to be crucial for understanding and mitigating CO_2_ emissions at a local level. This approach not only enhances precision in emissions monitoring but also lays a solid foundation for policy development, community engagement, and collaborative efforts towards global climate goals. Continued advancements in satellite technology, such as the upcoming Copernicus CO_2_ Monitoring Mission (CO2M), promise to enhance the spatial and temporal resolution of CO_2_ data, enabling more accurate and frequent monitoring of localized emissions^31^. Future research should prioritize improving the accuracy of satellite-based CO_2_ measurements, expanding the availability of ground-level CO_2_ data, and integrating comprehensive meteorological datasets. In particular, acquiring ground-truth data in urban, industrial, and non-European regions will be essential for validating and generalizing models trained within the ICOS network. Such efforts would greatly improve our ability to evaluate predictions in data-sparse regions and ensure the robustness of $\hbox {CO}_2$ monitoring systems at the global scale.

## Supplementary Information

Supplementary Information.

## Data Availability

The specific data points used during the current study are available from the corresponding author on reasonable request. However, all data used is publicly available for download from the following sources as well. 1. Era5 Weather - https://www.ecmwf.int/en/forecasts/dataset/ecmwf-reanalysis-v5 2. ICOS Ground level CO2 data - https://www.icos-cp.eu/data-products/ATM_NRT_CO2_CH4 3. OCO2 xCO2 data - https://disc.gsfc.nasa.gov/datasets?keywords=oco2.
