# Supplementary material for: Enhancing carbon emission reduction strategies using OCO and ICOS data
Source: Sci Rep. 2025 Oct 17;15:36297. doi: 10.1038/s41598-025-22022-1 (PMC12534651; doi:10.1038/s41598-025-22022-1)
Supplement: Supplementary file 1 — Supplementary Information. [file 41598_2025_22022_MOESM1_ESM.pdf]

Appendix: Model Hyperparameters

This paper considers three models in addition to the baseline prediction. The baseline model only uses the xCO_2 measurements from OCO as a naive prediction for the ICOS and therefore it has no hyperparameters.

Category Boosting

The Category Boosting model is based on the CatBoostClassifier model from the catboost package for python, using the following hyperparameters:

- $nbr\_classes = 25$
- $max\_depth = 6$
- $learning\_rate = 0.1$
- $iterations = 200$
- $l2\_leaf\_reg = 3$

Extreme Gradient Boosting

The Extreme Gradient Boosting model is based on the XGBRegressor model from the XGBoost package for python, using the following hyperparameters:

- $max\_depth = 6$
- $learning\_rate \quad (eta) = 0.05$
- $gamma = 0.9$
- $n\_estimators = 100$
- $lambda = 0$
- $alpha = 0.5$
- $subsamle = 50\%$
- $colsample\_bytree = 50\%$

Multilayer Perceptron

The multilayer perceptron model consists of 5 densely connected layers of sizes 64, 64, 128, 64 and 32 respectively. In addition, the model uses an output layer consisting of a single node with linear activation. A diagram of the MLP architecture is displayed in Figure 1.

With 14 input features, the number of total learnable parameters (weights and biases) was 23,809. This is calculated using the formula

$$\sum_{i=0}^{5}(n_i + 1) * n_{i+1} = 23809,$$

where n_i are the layer sizes (14,64,64,128,64, and 32).

In the internal layers of this neural network we employ a ReLU activation function which is one of the most widely used activation functions in neural networks, particularly in deep learning. It is defined as follows:

$$\text{ReLU}(x) = \max(0, x) = \begin{cases} x & \text{if } x \geq 0 \\ 0 & \text{if } x < 0 \end{cases}$$

Properties of ReLU:

- Non-linearity: ReLU introduces non-linearity to the model, which is crucial for learning complex patterns.
- Computationally efficient: It is simple to compute, which speeds up the training process.
- Sparse activation: Since the ReLU outputs zero for all negative inputs, it often leads to sparse activations, making the network more efficient.

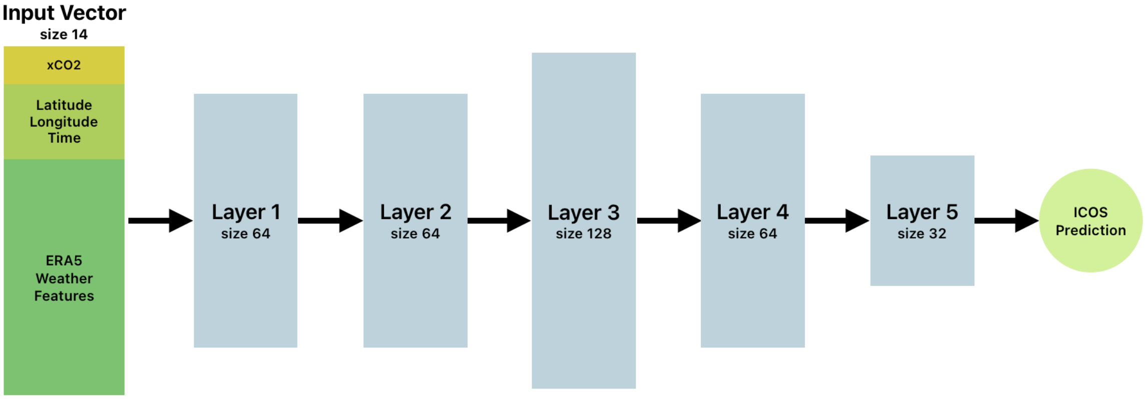

Figure 1. Diagram of MLP architecture with layer sizes included, along with the structure of the input vector and its sources.

We also employ L_2 regularization which is a technique used to prevent overfitting in neural networks by penalizing large weights in the model. The basic idea is to add a penalty term to the loss function, which discourages the model from learning excessively large weights. Furthermore, the model uses a learning rate of 0.001.

Given a loss function $\mathcal{L}(\mathbf{y}, \hat{\mathbf{y}})$, where $\mathbf{y}$ are the true labels and $\hat{\mathbf{y}}$ are the predicted labels, the L_2 regularized loss function is:

$$\mathcal{L}_{\text{reg}}(\mathbf{y}, \hat{\mathbf{y}}) = \mathcal{L}(\mathbf{y}, \hat{\mathbf{y}}) + \lambda \sum_i w_i^2$$

Here:

- $\mathcal{L}_{\text{reg}}$ is the regularized loss function.
- $\mathcal{L}$ is the original loss function (which in our case refers to mean squared error).
- w_i represents the weights of the model.
- λ is the regularization parameter (often called the regularization strength), which controls the amount of regularization applied.

The L_2 regularization term $\lambda \sum_i w_i^2$ is the sum of the squares of the weights, multiplied by the regularization parameter λ. For this network, $\lambda = 0.01$ was used. This term encourages the model to keep the weights small, which helps to reduce overfitting.
